# Supplementary material for: Predictors of Reactive Agility in Early Puberty: A Multiple Regression Gender-Stratified Study
Source: Children (Basel). 2022 Nov 19;9(11):1780. doi: 10.3390/children9111780 (PMC9688792; doi:10.3390/children9111780)
Supplement: Supplementary file 1 [file children-09-01780-s001.zip › Supplementary_Tables S1-S3.pdf]

Supplementary Table S1. Descriptive statistics for boys' sample (N=72)

Variable	X	SD	Minimum	Maximum	Max D	K-S
**AGE**	12.50	0.61	11.35	13.57	0.13	p < .20
**BH**	160.16	8.51	143.00	179.20	0.05	p > .20
**SH**	81.86	6.17	51.00	93.00	0.10	p > .20
**BM**	53.88	13.01	32.90	92.50	0.11	p > .20
**BF**	21.38	6.47	12.00	47.50	0.10	p > .20
**BJ**	154.22	28.60	79.00	213.00	0.07	p > .20
**S10**	2.16	0.19	1.85	2.71	0.11	p > .20
**S20**	3.88	0.40	3.18	5.14	0.12	p > .20
**20Y**	5.60	0.73	3.92	7.57	0.09	p > .20
**CODS**	2.86	0.40	2.18	4.03	0.14	p < .15
**TRAG**	3.54	0.52	2.10	5.02	0.09	p > .20
**SJ**	22.34	5.17	9.30	32.50	0.06	p > .20
**CMJ**	22.98	5.52	9.80	38.60	0.07	p > .20
**DJ**	21.57	5.81	0.00	33.90	0.07	p > .20
**RSI**	0.79	0.34	0.00	1.84	0.11	p > .20

Legend: BH – body height, **SH** - seated height, **BM** – body mass, **BF** – body fat, **BJ** – broad jump, **S10** – 10m sprint, **S20** – 20m sprint, **20Y** – 20 Yard shuttle agility test, **CODS** – change of direction, **TRAG** – "Triangle" reactive agility, **SJ** – squat jump, **CMJ** – countermovement jump, **DJ** – drop jump, **RSI** – reactive strength index

Supplementary Table S2. Descriptive statistics for girls' sample (N=58)

Variable	X	SD	Minimum	Maximum	Max D	K-S
**AGE**	12.44	0.53	11.34	13.40	0.08	p > .20
**BH**	160.46	7.40	140.00	177.20	0.09	p > .20
**SH**	86.34	18.03	70.00	181.00	0.09	p > .20
**BM**	52.87	11.73	32.70	80.40	0.09	p > .20
**BF**	24.47	5.56	16.30	39.50	0.19	p < .15
**BJ**	152.47	24.66	98.00	200.00	0.09	p > .20
**S10**	2.15	0.34	0.00	2.83	0.10	p > .20
**S20**	3.84	0.67	0.00	5.25	0.08	p > .20
**20Y**	5.78	0.77	3.96	8.79	0.16	p < .10
**CODS**	2.95	0.39	2.36	4.23	0.11	p > .20
**TRAG**	2.96	0.39	2.36	4.24	0.11	p > .20
**SJ**	3.72	0.58	2.10	5.92	0.11	p > .20
**CMJ**	21.10	6.13	0.00	31.00	0.06	p > .20
**DJ**	22.15	6.18	0.00	34.60	0.07	p > .20
**RSI**	20.91	5.69	0.00	31.50	0.08	p > .20

Legend: BH – body height, **SH** - seated height, **BM** – body mass, **BF** – body fat, **BJ** – broad jump, **S10** – 10m sprint, **S20** – 20m sprint, **20Y** – 20 Yard shuttle agility test, **CODS** – change of direction, **TRAG** – "Triangle" reactive agility, **SJ** – squat jump, **CMJ** – countermovement jump, **DJ** – drop jump, **RSI** – reactive strength index

Supplementary Table S3. T-test for independent samples between boys and girls

Variable	t-value	df	p
**AGE**	-0,55	129	0,58
**BH**	0,05	129	0,96
**SH**	1,16	129	0,25
**BM**	-0,52	102	0,60
**BF**	2,44*	101*	0,01*
**BJ**	-0,15	129	0,88
**S10**	0,53	129	0,59
**S20**	-0,04	129	0,97
**20Y**	0,68	129	0,50
**CODS**	0,81	129	0,42
**TRAG**	1,47	129	0,14
**SJ**	-1,02	129	0,31
**CMJ**	-0,59	129	0,55
**DJ**	-0,42	129	0,67
**RSI**	0,06	129	0,95

Legend: BH – body height, **SH** - seated height, **BM** – body mass, **BF** – body fat, **BJ** – broad jump, **S10** – 10m sprint, **S20** – 20m sprint, **20Y** – 20 Yard shuttle agility test, **CODS** – change of direction, **TRAG** – "Triangle" reactive agility, **SJ** – squat jump, **CMJ** – countermovement jump, **DJ** – drop jump, **RSI** – reactive strength index
